# Epidemiology of foot burns in a Dutch burn centre

**DOI:** 10.1186/s41038-015-0003-y

**Published:** 2015-06-15

**Authors:** Tülay Kılıç, Pieta Krijnen, Wim E Tuinebreijer, Roelf S Breederveld

**Affiliations:** 1 Department of Surgery, Leiden University Medical Center University Leiden, 2300 RC Leiden, The Netherlands; 2 Acute Burn Care & Trauma Surgery, Department Surgery/Burn Centre, Rode Kruis Ziekenhuis, Vondellaan 13, 1942 LE Beverwijk, The Netherlands

## Abstract

**Background:**

Although the feet involve a small percentage of the total body surface area, they can have major effects in daily life, caused by prolonged bed rest, hospitalization and high risk of both early and late complications. The aim of this study was to define the aetiology, treatment and outcomes of foot burns, with special interest in paediatric patients, patients with diabetic disease and burns acquired at the workplace.

**Methods:**

This is a retrospective cohort study of 82 patients who were admitted to one of the three burn centres in the Netherlands during the period 2004 to 2013. The patients had a median age of 43.5 years (range 0.01–85.9), and included 14 children and 8 diabetics. Data were collected from the hospital records.

**Results:**

Scalding was the most common cause of the injury. Almost all patients required surgical management. It is notable that most of hospitalized patients (82 %) were not admitted on the day of injury. Children had a significantly shorter length of stay compared to adults (*p* = 0.01). The eight patients with diabetes had a significantly longer length of hospital stay, more complications and more often residual defects compared to the non-diabetic patients. In 13 patients, the injury took place at work. Half of these burns were caused by scalding, and foot burns caused by chemicals at work were rare (two patients).

**Conclusions:**

Although the incidence of foot burns is low, there is a significant morbidity due to complications and long hospital stay. The following three points are suggested. Immediate referral to a burn centre is essential. It is important to educate diabetic patients on the risk of complications and adverse outcomes after burn injury. Preventative measures at the workplace are worth considering.

## Background

Although the feet involve only a small percentage of the total body surface area (3.5 %), they play an important role in daily life because of their motion and weight-bearing function. Burns to the feet can have major effects in daily life, caused by prolonged bed rest, hospitalization and high risk of both early and late complications [1].

Children, elderly patients and patients with diabetes and neurological disorders form risk groups for foot burn injuries [1]. There have been studies from various countries about foot burns in diabetic patients and children [1-13], but only one about foot burns in diabetic patients was performed in the Netherlands [11]. These studies show that aetiology, treatment and outcomes may differ [1-6,12-14].

The purpose of this study was to describe the aetiology, treatment and outcomes of foot burns in a burn centre in the Netherlands, with a special interest in the known high-risk groups of children and patients with diabetes. The foot burns acquired at the workplace were also examined, because it was expected that accidents with chemicals at work may also form a specific risk group that may be addressed with preventive measures.

## Methods

In this retrospective cohort study, discharge letters containing various terms and synonyms for “foot” and “burns” were selected and examined of patients who were admitted to one of the three burn centres in the Netherlands during the period February 2004 to March 2013. After reviewing the letters, patients who had foot burns or burns on the foot and lower leg, were selected. Patients with more severe burn wounds (burn depth and % total body surface area (TBSA)) in other body regions were excluded, because in these patients the healing process was mainly determined by the burn wounds located in other body parts.

The following data were collected from the hospital records: age, gender, comorbidities, location of accident, date of burn, date of hospital admission, date of discharge, percent total body surface area burned (% TBSA burned), burn agent, depth of burn, anatomic location of the burn, complications, treatment type, take of graft, date of complete epithelialization and suspected child abuse. In the study, hospitalization was defined as admission of more than 1 day in hospital. Complete epithelialization was defined as the complete coverage of the burn wound.

The following groups were compared regarding patient characteristics, injury characteristics, complications and outcome: children (<18 years) versus adults, diabetics versus non-diabetics and groups with different locations of the accident.

### Data analysis

Standard statistical techniques were used to analyse differences between the groups: the Mann–Whitney test for skewed continuous variables and the chi-squared or Fisher’s exact test for categorical variables as appropriate. All analyses were performed using the Statistical Package for the Social Sciences (version 22; SPSS Inc., Chicago, IL).

## Results

### Study population

Two hundred and forty-six discharge letters of patients treated in the study period contained the terms “foot” and “burns” or similar terms. After examining these letters, 164 patients were excluded from the analyses because they did not meet the inclusion criteria.

The remaining 82 patients (14 children and 68 adults) with foot burns were seen at the burn centre during a 9-year study period, which means an average incidence of nine patients with foot burns per year.

The median and mean age (standard deviation (SD)) of the study population was, respectively, 43.5 years (range 0.01–85.9) and 40 years (SD 21), with more male than female patients (2.3:1). Of the adult patients, 45 (66.2 %) were men, and from the paediatric patients, 12 (85.7 %) were boys. The comorbidity of the adult patients consisted of eight (11.8 %) patients with DM, four (5.9 %) with neuropathy, one (1.5 %) with spina bifida, two (2.9 %) with peripheral vascular disease, two (2.9 %) with cerebral palsy and one (1.5 %) with mental retardation. One (7.1 %) of the paediatric patients suffered from spina bifida. Therefore, many of the adult patients have diminished feeling in their feet.

The patients were usually admitted to hospital (79 %) after a median period of 14 days (range 0–49) after trauma. Seventeen patients were treated and sent home within 1 day. The other 65 patients who were hospitalized for at least 1 day had an average hospital length of stay of 9 days (range 1–41). Twelve of these hospitalized patients (18 %) were admitted to hospital within 24 h after the accident, and 53 patients (82 %) were admitted to hospital after 1 day or more.

Complications developed in two of the patients (17 %) who were admitted on the day of injury and in 15 patients (28 %) who were admitted more than 24 h after the injury (*p* = 0.674).

Nineteen (34.5 %) of the adult patients were smokers. The period till complete epithelialization was 76.8 days (SD = 51.6) in the smokers compared with 30.2 days (SD = 3.1) for the non-smokers (*p* = 0.001).

### Comparison between patient groups

#### Adult and paediatric group

The study group consisted of 68 adult and 14 paediatric patients. The median and mean age (SD) of the adult population was, respectively, 46.0 years (range 18.7–85.9) and 46.7 years (15.6) and of the children, respectively, 4.7 years (range 0.01–14.9) and 5.9 years (5.2). Comorbidity was rare in the paediatric patients, and diabetes mellitus was the most common comorbidity in the adult group (eight patients). In the adult patients, 13 (19.1 %) feet burns were caused by an accident at the workplace, and 3 burns (4.4 %) were due to automutilation. In the paediatric group, there was one case of suspected child abuse (7 %).

Scald burns were the most common cause of burn in both groups with a percentage of 52.9 % in adults and 64.3 % in children (Table 1). While chemical burns followed scald burns in the adult group, contact and firework burns were the second most common in the paediatric group.

**Table 1:** Burn characteristics of 82 patients with foot burns by age group

		Adult patients (*N* = 68)	Paediatric patients (*N* = 14)
Burn agent, No (%)	Scald	36 (52.9)	9 (64.3)
	Flame	5 (7.4)	0 (0.0)
	Contact	4 (5.9)	2 (14.3)
	Chemical	7 (10.3)	0 (0.0)
	Firework	3 (4.4)	2 (14.3)
	Ash/coals	3 (4.4)	1 (7.1)
	Other	10 (14.7)	0 (0.0)
Burn depth, No (%)^a^	Second degree	10 (14.9)	3 (23.1)
	Third degree	21 (31.3)	5 (38.5)
	Mixed second and third degree	34 (50.7)	5 (38.5)
	Mixed third and fourth degree	2 (3.0)	0 (0.0)
% TBSA, median (range)^a^		1.5 (0.5–11.5)	1.0 (0.5–12.0)
Surface, No (%)^b^	Dorsal	40 (71.4)	8 (66.7)
	Plantar	4 (7.1)	2 (16.7)
	Both	12 (21.4)	2 (16.7)
Side, No (%)	Right	24 (35.3)	5 (35.7)
	Left	14 (20.6)	5 (35.7)
	Both	30 (44.1)	4 (28.6)

^a^Missing for one adult and one paediatric patient

^b^Missing for 12 adults and 2 paediatric patients

Patients in the adult group had generally mixed second- and third-degree burns (50.7 %), whereas in children third-degree and mixed second- and third-degree burns were equally common (38.5 %). The majority of both adults and children had burns on a single foot (Table 1). Both feet were more frequently involved in adult patients. In adult and paediatric patients, the dorsal side of the feet was most frequently injured (Table 1).

Time till admission lasted not significantly longer in adult patients (Table 2). Not significantly more adult patients compared to paediatric patients were admitted to hospital. None of the children were readmitted compared to nine (13 %) of the adult patients (*p* = 0.34). It is notable that the length of stay was shorter in the paediatric group than in the adult group (*p* = 0.01) (Table 2).

**Table 2:** Outcomes in 82 patients with foot burns by age group

	Adult patients (*N* = 68)	Paediatric patients (*N* = 14)	*p* value
Days till admission, median (range)	14.5 (0–49)	11 (0–17)	0.095
Referral, No (%)			0.141
Admission to hospital > 1 day	56 (82.4)	9 (64.3)	
Admission to hospital refused by patient/parents	1 (1.5)	1 (7.1)	
Day treatment	11 (16.2)	4 (28.6)	
Readmittance to hospital, No (%)^a^	9 (13.4)	0 (0.0)	0.342
Median length of stay, days (range)	6.0 (1–41)	5.0 (1–7)	0.012
Treatment^b^			
			0.065
Graft	61 (91.0)	10 (71.4)	
Necrotectomy only	1 (1.5)	0 (0.0)	
Conservative	5 (7.5)	3 (21.4)	
Other	0 (0.0)	1 (7.1)	
Additional surgery, No (%)^b^	6 (9.0)	0 (0.0)	0.583
Take graft, No (%)^c^			0.240
Good	54 (84.4)	10 (76.9)	
Moderate	5 (7.8)	0 (0.0)	
Inapplicable	5 (7.8)	3 (23.1)	
Complete epithelialization, No (%)^e^	51 (83.6)	10 (83.3)	1.000
Residual defect, No (%)^d^	21 (32.8)	2 (16.7)	0.327
Days till complete epithelialization, median (range)	31 (10–192)	29.5 (24–33)	0.613
Complications, No (%)^f^	15 (23.1)	3 (27.3)	0.716

^a^Missing for one adult and one paediatric patient

^b^Missing for one adult patient

^c^Missing for four adults and one paediatric patient. Good: >90 %; moderate: 50–90 %; inapplicable: <50 %

^d^Missing for four adults and two paediatric patients

^e^Missing for seven adults and two paediatric patients

^f^Missing for three adults and three paediatric patients. Infection, graft loss, delayed wound healing, hypertrophic scaring, scar contracture, amputation and shock

Most of the patients in both groups underwent surgery. Grafting was nearly always preceded by necrotectomy, and in only one adult patient, necrotectomy was the only intervention. There were no significant differences between the groups regarding outcome (complete epithelialization, residual defects, days till complete epithelialization and complications) (Table 2).

#### Presence of diabetes in adult patients

Scalding was the most common cause of foot burn among both diabetic and non-diabetic patients, in 62.5 and 51.7 %, respectively. There were no significant differences between diabetic and non-diabetic patients regarding % TBSA burned, burn depth, side and affected surface. Neuropathy was more common in diabetic patients (37.5 versus 1.4 %, *p* = 0.002).

Time till admission lasted not significantly longer in non-diabetic patients, and not significantly more diabetic patients compared to non-diabetic were admitted to hospital (Table 3). Additional hospitalization was not more often necessary in diabetic patients. Diabetic patients had a significantly longer length of hospital stay and more often complications compared to the non-diabetic patients (Table 3). The following complications were more common in diabetes: infection, graft loss, delayed wound healing and scar contraction, but these subgroups were too small to show statistically significant differences.

**Table 3:** Burn characteristics and outcome in adults by presence of diabetes

	Diabetic (*N* = 8)	Non-diabetic (*N* = 60)	*p* value
Days till admission, median (range)	4 (0–49)	15 (0–49)	0.367
Referral, No (%)			0.088
Admission to hospital > 1 day	7 (87.5)	49 (81.7)	
Admission to hospital refused by patient	1 (12.5)	0 (0.0)	
Day treatment	0 (0.0)	11 (18.3)	
Additional hospitalization, No (%)^a^	1 (12.5)	8 (13.6)	1.000
Median length of stay, days (range)	20 (4–41)	5 (1–30)	0.025
Complications, No (%)^b^	5 (62.5)	10 (17.5)	0.013
Treatment, No (%)^a^			0.549
Graft	7 (87.5)	54 (91.5)	
Necrotectomy only	0 (0.0)	1 (1.7)	
Conservative	1 (12.5)	4 (6.8)	
Additional surgery, No (%)^a^	2 (25.0)	4 (6.8)	0.147
Complete epithelialization after first treatment, No (%)^c^	6 (85.7)	45 (83.3)	1.000
Days till complete epithelialization, median (range)	44 (17–93)	31 (10–192)	0.660
Take graft, No (%)^d^			
			0.365
Good	6 (75.0)	48 (85.7)	
Moderate	1 (12.5)	4 (7.1)	
Inapplicable	1 (12.5)	4 (7.1)	

^a^Missing for one non-diabetic patient

^b^Missing for three non-diabetic patients. Infection, graft loss, delayed wound healing, hypertrophic scaring, scar contracture, amputation and shock

^c^Missing for one diabetic patient and six non-diabetic patients

^d^Missing for four non-diabetic patients

Most of the patients in both groups underwent surgery. There were no significant differences in intervention type. Grafting was nearly always preceded by necrotectomy, and in only one non-diabetic patient, necrotectomy was the only intervention. There were no significant differences between the groups regarding outcome (additional surgery, percentage of and days till complete epithelialization and percentage take graft) (Table 3).

#### Place of injury in adults: workplace and other

Thirteen of the foot burn injuries took place at work. Scald burns was the most common cause (*N* = 7, 53.8 %). Foot burns at the workplace caused by chemicals occurred in only two patients (15.4 %). For the other cases, the mechanism of trauma was running of hot liquid in shoes in six patients, stepping accidentally in hot water by three patients, one accident with a cutting torch and unknown for one case. There were no significant differences in characteristics of the burn like % TBSA burned and depth and location (side and surface) between foot burns caused in the workplace and those caused in other situations (Table 4).

**Table 4:** Burn characteristics and outcome in adults by place of injury

	Workplace (*N* = 13)	Other (*N* = 55)	*p* value
% TBSA, median (range)^a^	3.0 (0.5–6.0)	1.0 (0.5–11.5)	0.055
Burn depth, No (%)^a^			0.374
Second degree	4 (30.8)	6 (11.1)	
Third degree	3 (23.1)	18 (33.3)	
Mixed second and third degree	6 (46.2)	28 (51.9)	
Mixed third and fourth degree	0 (0.0)	2 (3.7)	
Burn agent, No (%)			0.513
Scald	7 (53.8)	29 (52.7)	
Flame	0 (0.0)	5 (9.1)	
Contact	0 (0.0)	4 (7.3)	
Chemical	2 (15.4)	5 (9.1)	
Firework	0 (0.0)	3 (5.5)	
Ash/coals	0 (0.0)	3 (5.5)	
Other	4 (30.8)	6 (10.9)	
Surface, No (%)^b^			0.719
Dorsal	7 (70.0)	33 (71.7)	
Plantar	0 (0.0)	4 (8.7)	
Both	3 (30.0)	9 (19.6)	
Side, No (%)			0.665
Right	4 (30.8)	20 (36.4)	
Left	4 (30.8)	10 (18.2)	
Both	5 (38.5)	25 (45.5)	

^a^Missing for one patient with other place of injury

^b^Missing for nine patients with injury in the workplace and three patients with other place of injury

## Discussion

The purpose of this study was to estimate the incidence of foot burns in a burn centre in the Netherlands and to gain information about aetiology, characteristics and outcome of patients and injury.

The average incidence of nine patients with foot burns per year in the burn centre was low. The burn centre is one of the three burn centres in the Netherlands. In the Netherlands, patients with superficial burns frequently visit their general practitioner and get treatment at the general practice. This may explain why relatively few patients were seen in the burn centre. This may also explain the large number of patients with deep injuries and the high percentage of grafting procedure (88 %) in our study compared with other studies (25–29 %) [1,14].

The aetiology of foot burns varies in the literature. Hemington-Gorse et al. and Zachary et al. [1,14] found scalding as the most common cause of foot burns, which is comparable with our study. In children, contact burns were the most common cause of the injury in countries with a warm climate, like Australia, South Africa and Central Asia, where walking or standing barefoot on hot ground caused foot burns [2-6,12,13]. Our study confirms the finding of a study in the United Kingdom [1] that in countries with a temperate climate scalding is the most common cause of foot burns in children. In the paediatric group, there was one case of suspected child abuse (7%) based on the history and presentation of the patient. In this case, the in-hospital protocol was activated, but after contact with the parents of the child, the abuse was not proven nor admitted. There is no literature about foot burns due to child abuse. Ruth et al. mentioned that up to 20 % of all admitted paediatric burn patients were the victims of child abuse or neglect [15].

In our study, complications occurred in 18 (22 %) of the patients of whom 7 patients had more than 1 complication. Although this outcome is comparable with a British study (18 %) [1], the most common complication was different from this study. While the most common complication was infection in our study, hypertrophic scarring was the most common complication in the British study [1]. This difference may be caused by more conservative treatment. In the study of Hennington-Gorse et al. [1], 75 % of the patients were treated with dressings whereas 87 % of our patients were treated with skin grafting. Zachary et al. [14] examined only complications like hypertrophic scarring and range of motion loss which occurred, respectively, in 5 and 6 % in their whole study population. In our study, 3.7 % of the patients had hypertrophic scarring as a complication, which was subjectively determined by the doctor who saw the patient in the outpatient clinic. Foot burns are not prone to hypertrophic scarring, and our treatment with skin grafting could provoke a positive effect on hypertrophy. The increased rate of residual defects, especially in adults (33 %), was not a big problem because the take rate of the grafts was good in 84 %, and additional surgery was necessary in 9 % of the adult patients.

While most of the patients (83 %) were not admitted on the day of injury, patients who were admitted on the day of injury seemed to have fewer complications than patients who were admitted later, but the difference was not significant.

The total number of patients with foot burns includes 14 children and 68 adults. Of the adult patients, 13 (19 %) had acquired the injury at the workplace, and 18 (26 %) had comorbidity with most frequently diabetes. Comparison between our defined groups showed relevant differences. In the first place, there was significant difference in length of stay between adult and paediatric group, while there was no significant difference in burn depth. This may be the result of the presence of comorbidity in the adult group.

In the second place, there were significant differences in complications, median length of stay and rest defects between diabetic and non-diabetic patients. This is partially comparable with other studies on foot burns in diabetic patients that have reported that foot burns in patients with diabetes are deeper and associated with worse outcomes such as higher rates of infection, graft failure, more operations and longer hospital stay [7-11].

In the third place, the percentage of patients who acquired foot burns at work in our study (19 %) was lower compared with the study of Hemington-Gorse et al. (47 %) [1]. The study of Hemington-Gorse et al. was performed in a region with a number of large chemical industrial units and many local manual workers [1]. This may explain the difference in the proportion of work-related foot burn injuries between the studies. The results of this paper may be used for preventing foot burns in high-risk groups and may be useful for the prediction of a clinical course of foot burns, which is relevant when informing patients about complications and other outcomes.

A major limitation of this study is the small study population. Also, many differences between groups were evaluated, which may have led to false-positive findings due to multiple testing. Missing data was another limitation because of the retrospective design of this study.

## Conclusions

Although the incidence of foot burns is low, there is a significant morbidity due to complications and long hospital stay. The following points are suggested:

- The immediate referral to a burn centre is essential due to the high-complication rate.
- Because of the high-complication rate in diabetic patients, it is important to educate these patients on the risk of complications and adverse outcomes after burn injuries and raise awareness that immediate referral to a burn centre is essential.
- One in five of the adult patients acquired their foot burn injury at work; therefore, preventative measures at the workplace are worth considering as protective pants that cover the entrance of the shoe.

## Abbreviations

TBSA: Total body surface area
SPSS: Statistical Package for the Social Sciences
SD: Standard deviation
No: Number
